# Molecular diet analysis finds an insectivorous desert bat community dominated by resource sharing despite diverse echolocation and foraging strategies

**DOI:** 10.1002/ece3.4896

**Published:** 2019-02-23

**Authors:** Rowena Gordon, Sally Ivens, Loren K. Ammerman, M. Brock Fenton, Joanne E. Littlefair, John M. Ratcliffe, Elizabeth L. Clare

**Affiliations:** ^1^ School of Biological and Chemical Sciences Queen Mary University of London London UK; ^2^ Department of Biology Angelo State University San Angelo Texas USA; ^3^ Department of Biology University of Western Ontario London Ontario Canada; ^4^ Department of Biology University of Toronto Mississauga Mississauga Ontario Canada; ^5^ Department of Biology McGill University Montréal Québec Canada

**Keywords:** bat foraging ecology, community ecology, dietary analysis, metabarcoding

## Abstract

Interspecific differences in traits can alter the relative niche use of species within the same environment. Bats provide an excellent model to study niche use because they use a wide variety of behavioral, acoustic, and morphological traits that may lead to multi‐species, functional groups. Predatory bats have been classified by their foraging location (edge, clutter, open space), ability to use aerial hawking or substrate gleaning and echolocation call design and flexibility, all of which may dictate their prey use. For example, high frequency, broadband calls do not travel far but offer high object resolution while high intensity, low frequency calls travel further but provide lower resolution. Because these behaviors can be flexible, four behavioral categories have been proposed: (a) gleaning, (b) behaviorally flexible (gleaning and hawking), (c) clutter‐tolerant hawking, and (d) open space hawking. Many recent studies of diet in bats use molecular tools to identify prey but mainly focus on one or two species in isolation; few studies provide evidence for substantial differences in prey use despite the many behavioral, acoustic, and morphological differences. Here, we analyze the diet of 17 sympatric species in the Chihuahuan desert and test the hypothesis that peak echolocation frequency and behavioral categories are linked to differences in diet. We find no significant correlation between dietary richness and echolocation peak frequency though it spanned close to 100 kHz across species. Our data, however, suggest that bats which use both gleaning and hawking strategies have the broadest diets and are most differentiated from clutter‐tolerant aerial hawking species.

## 1 INTRODUCTION

Studies of trophic interactions between species increase our understanding of how intrinsic and extrinsic characteristics structure resource use in communities (Arrizabalaga‐Escudero et al., 2018), how populations respond to resource limitations, and what impact these responses have on ecological processes (Amarasekare, 2008; McCann, 2007; Milo et al., 2002; Rooney & McCann, 2012). Niche is an N‐dimensional concept, and thus impossible to quantify, but a number of key niche indicators are measured regularly. Diet is one of the most obvious and common but can be difficult to assess in detail when the resource base is diverse and the behavior of the consumer is cryptic (Clare, 2014). Molecular analyses of gut or fecal contents have a long methodological history (Symondson, 2002) and are now becoming common, particularly when tied to high‐throughput sequencing (HTS) technologies (Clare, Symondson, & Fenton, 2014; Nielsen, Clare, Hayden, Brett, & Kratina, 2018; Pompanon et al., 2012).

Insectivorous bats have been a frequent target for molecular dietary analyses (e.g., Bohmann et al., 2011; Clare et al., 2014; Razgour et al., 2011). The cryptic nature of bat behavior (flight, nocturnal activity) makes them a challenge for observation, but quick digestive transit times and communal roosts mean fecal samples can be collected from bats quickly and molecular approaches have made the investigation of their diets a tractable issue. Understanding which intrinsic characteristics bats employ to acquire prey and how trait variation may influence this is of considerable interest as bats occur in great numbers and at high taxonomic diversity even in resource‐poor habitats, often co‐occurring in large multi‐species assemblages (Ammerman, Schmidly, & Hice, 2012; Kunz, Braun de Torrez, Bauer, Lobova, & Fleming, 2011). Some authors have argued for partitioning of dietary resources along species lines with echolocation and behavior as the main drivers of prey use (Denzinger, Tschapka, & Schnitzler, 2017; Schnitzler & Kalko, 2001), while others (Kunz & Fenton, 2005; Willis, Voss, & Brigham, 2006) have suggested alternative resources, such as available roosts, are more limiting. Evidence from molecular studies has found a consistent pattern of overlapping use of diet within bat communities and a number of studies have suggested other factors such as spatial (Arita, 1991) or temporal separation of hunting activity (Emrich, Clare, Symondson, Koenig, & Fenton, 2014) or selection of prey by life stages (Krüger et al., 2014) as potential mechanisms of niche partitioning that may not lead to observable dietary differences from the analysis of feces. A limitation of these analyses has been that most focus on one or a few species in isolation and none have considered communities as a whole. Thus, assessments of how intrinsic characteristics might shape resource use across a community are rare in the molecular diet analysis literature.

One reason why bats are an ideal model for this question is that they exhibit a wide variety of behavioral, acoustic, and morphological traits which are thought to combine to form discrete, multi‐species, functional groups. Characteristics used to group bats in guilds have included (a) open space, narrow space, or edge foraging (Schnitzler, Moss, & Denzinger, 2003), (b) aerial hawking (taking prey on the wing) or substrate gleaning (taking prey from surfaces) (Clare & Holderied, 2015; Norberg & Rayner, 1987), and (c) echolocation call design and flexibility, where variation in call frequency, structure, duty cycle (Jones, 1999) and intensity (Surlykke & Kalko, 2008) could dictate their ability to find and capture prey. For example, high frequency, broadband calls do not travel far in open spaces but offer high object resolution (Jones, 1999) and are correlated with short, broad wings which allow maneuverable flight (Denzinger & Schnitzler, 2013). High intensity, low frequency calls over a smaller bandwidth travel further but provide lower resolution (Denzinger & Schnitzler, 2013; Jones, 1999). As such, bats described by the former traits are thought to be better able take prey found in cluttered environments (e.g., edges) and the latter prey in open areas (Fenton, 1990) potentially impacting dietary niche. Because these behaviors can be flexible, Ratcliffe, Fenton, and Shettleworth (2006) proposed four behavioral categories for predatory bats: (a) gleaning bats, (b) behaviorally flexible bats (gleaning and aerial hawking), (c) clutter‐tolerant aerial hawking bats, and (d) open space aerial hawking bats.

**Figure 1:**
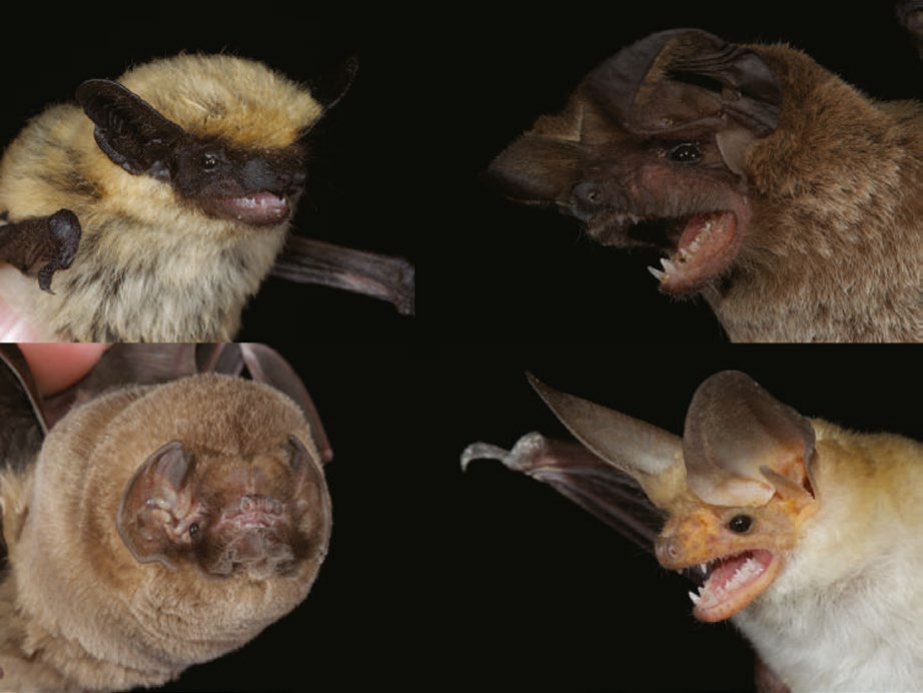
A variety of bat species co‐occur in Big Bend National Park (Texas, USA). Clockwise from top left ‐ *Parastrellus hesperus, Nyctinomops femorosaccus, Antrozous pallidus, and Mormoops megalophylla*

Here, we undertook a molecular dietary analysis of a whole community of sympatric bats rather than one including just one or two species. We focus on insectivorous bats in Big Bend National Park (S.W. Texas, USA, Figure 1). This location is a protected area of the Chihuahuan Desert that supports a community of at least 19 species of bats (Easterla, 1973a, 1973b; Higginbotham, Ammerman, & Dixon, 1999) with very diverse echolocation call designs and hunting styles (Ammerman et al., 2012). This allows us to examine the role of these intrinsic traits in prey resource use in a community context. In this analysis, we scale up from previous investigations by considering almost the entire bat community (*N* = 17 species) including species of all four categories of foraging behavior suggested by Ratcliffe et al. (2006) and a range of peak echolocation call frequencies from an extraordinarily low 8 kHz to a maximum of more than 90 kHz.

Several predictions arise from these methods of ecologically classifying bats into functional groups; for example, that assigned behavioral category and peak echolocation call frequency will influence diet, niche breadth and overlap. Here, we use HTS to analyze the diet of 17 co‐occurring species from samples collected across seasons and years. Using these data we assess: (a) the size and richness of dietary niche for each species, (b) the degree of niche overlap between species, and (c) the relationship between behavior, echolocation peak frequency and diet.

## 2 MATERIALS AND METHODS

### 2.1 Study site and sample collection

**Figure 2:**
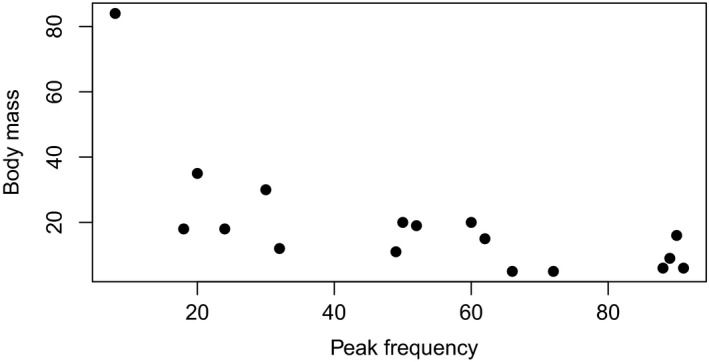
Largest recorded body mass (g) versus peak echolocation frequency (kHz) for 17 species of bat in Big Bend National Park Texas. Among vespertilionids such as the *Myotis* with short call durations, lower peak frequency is associated with higher wing loading and aspect ratio (Norberg & Rayner, 1987). Comparatively, molossids have longer calls of narrower bandwidth (Jung, Molinari, & Kalko, 2014). Among most species in our study, peak frequency increases as body mass decreases. *Eumops perotis* stands out as being both exceptionally large and low frequency. Body mass estimates were taken from Ammerman et al. (2012). For peak frequencies see Table 1

We obtained fecal (guano) samples collected from 309 individuals of 17 species of bats (Tables 1 and 2, Figure 2) from Big Bend National Park from 2011 to 2015. Almost all samples were collected in May and June with a small number from April or July to augment sample sizes of rare species. In addition, 5 samples collected from *Eumops perotis* were included from March of 2003. Year‐round, the conditions of Big Bend can be harsh with 31.7 cm annual rainfall ranging from around 0.737 cm in March to 5.41 cm in August and temperatures ranging from 35.1°C in June to 16.4°C in January (www.nps.gov/bibe/planyourvisit/weather.htm). Bats were captured by mist netting and each was identified to species level and then kept in a fabric bag or paper cup for approximately an hour, so that fecal samples could be collected before the bats were released. Gut transit times for insectivores can be fast (≈30 min in small bats, longer in larger species e.g., ≈120 min in *Eptesicus fuscus*) (Buchler, 1975) so a one‐hour wait period is a reasonable time to expect the most recent meal to have been digested and excreted. Fecal samples were stored in sterile tubes and frozen, desiccated or preserved in 96% ethanol (depending on sampling campaign).

**Table 1:** Behavioral classification of bats in Big Bend National Park Texas (US) based on the categories of Ratcliffe et al. (2006) where (1) gleaning bats, (2) behaviorally flexible bats (gleaning and aerial hawking), (3) clutter‐tolerant aerial hawking bats, and (4) open space aerial hawking bats. Peak reported echolocation frequencies are given

Species	Samples Collected	Behavioral Category	Peak Frequency (kHz)	Echolocation reference
*Antrozous pallidus*	27	1	60	Measor et al. (2017)
*Corynorhinus townsendii*	22	2	32	Corcoran and Conner (2017)
*Eptesicus fuscus*	4	4	50	Fullard and Dawson (1997)
*Euderma maculatum*	1	4	24	Fullard and Dawson (1997)
*Eumops perotis*	10	4	8	E.L. Clare unpublished data
*Lasiurus cinereus*	1	4	20	Barclay (1986)
*Mormoops megalophylla*	17	4	52	Rydell, Arita, Santos, and Granados (2002)
*Myotis californicus*	22	3	72	Gannon, Sherwin, Decarvalho, and O'Farrell (2001)
*Myotis ciliolabrum*	11	3	66	Gannon et al. (2001)
*Myotis thysanodes*	33	2	49	Fenton and Bell (1981)
*Myotis velifer*	17	3	90	Thomas, Bell, and Fenton (1987)
*Myotis volans*	2	3	89	Fenton and Bell (1981)
*Myotis yumanensis*	22	3	88	Thomas et al. (1987)
*Nyctinomops femorosaccus*	26	4	18	Ammerman et al. (2012)
*Nyctinomops macrotis*	4	4	30	www.sonobat.com
*Parastrellus hesperus*	55	3	91	Fenton and Bell (1981)
*Tadarida brasiliensis*	34	4	62	Fenton and Bell (1981)

**Table 2:** Dietary richness of bat species based on MOTU counts for each order of arthropods

	*n*	Diptera	Lepidoptera	Hemiptera	Coleoptera	Aranaea	Hymenoptera	Ephemeroptera	Neuroptera	Orthoptera	Psocoptera	Trichoptera	Collembola	Trombidiformes	Oribatida	Chordeumatida	Odonata	Total MOTU	Total Orders
*Myotis thysanodes*	32	133	83	4	14	6	5	14	2	4	1	2	3	0	1	1	0	273	14
*Eptesicus fuscus*	3	22	16	1	1	1	2	1	1	1	1	0	0	0	0	0	0	47	10
*Parastrellus hesperus*	39	37	76	22	7	1	2	6	1	0	1	0	0	0	1	0	0	154	10
*Corynorhinus townsendii*	19	101	12	2	4	4	3	0	0	0	0	4	1	1	0	0	0	132	9
*Myotis ciliolabrum*	9	40	25	1	2	4	2	0	0	0	0	1	0	1	0	1	0	77	9
*Myotis yumanensis*	21	31	94	10	11	8	0	8	0	2	0	1	1	0	0	0	0	166	9
*Mormoops megalophylla*	17	10	153	2	1	1	0	0	4	10	1	0	0	0	0	0	0	182	8
*Myotis velifer*	16	71	67	2	4	0	4	0	1	0	0	0	2	1	0	0	0	152	8
*Nyctinomops femorosaccus*	24	65	231	14	6	2	2	0	2	0	0	1	0	0	0	0	0	323	8
*Antrozous pallidus*	26	22	36	3	0	0	0	2	0	1	1	0	0	0	1	0	0	66	7
*Eumops perotis*	10	21	80	1	0	0	0	6	0	4	0	0	0	0	1	0	2	115	7
*Tadarida brasiliensis*	32	20	286	32	15	0	2	6	4	0	0	0	0	0	0	0	0	365	7
*Myotis californicus*	13	2	70	2	1	5	0	0	0	0	0	0	0	0	0	0	0	80	5
*Nyctinomops macrotis*	4	3	75	1	0	0	0	0	0	0	1	0	0	0	0	0	0	80	4
*Lasiurus cinereus*	1	6	0	0	0	1	0	0	0	0	0	0	0	1	0	0	0	8	3
*Myotis volans*	2	9	5	0	0	0	0	0	0	0	0	0	0	0	0	0	0	14	2
*Euderma maculatum*	1	0	1	0	0	0	0	0	0	0	0	0	0	0	0	0	0	1	1

Note

Shading indicates the presence or absence of the order in the diet while the values indicate the number of MOTU differentiated.

### 2.2 Insect DNA recovery and processing

We extracted insect DNA from guano using the QIAamp DNA stool mini kit (Qiagen UK) with modifications as suggested by Zeale, Butlin, Barker, Lees, and Jones (2011) and Clare et al. (2014). The eluted DNA was transferred to 96 well plates and sent for PCR and sequencing at the Biodiversity Institute of Ontario, University of Guelph (Canada). We PCR amplified the regions described by Zeale et al. (2011) using primers modified for the IonTorrent platform as described by Clare et al. (2014). These primers do not easily amplify mammal DNA when used in metabarcoding of mixed templates and thus Chiropteran DNA is rarely amplified or sequenced in more than trace quantities. Similarly, bacterial DNA is not easily amplified using this protocol. We used a dual index system with unique molecular identification tags (MIDs) on both forward and reverse primers. For each 20 µl PCR reaction, we used 10 µl of Qiagen multiplex PCR (Qiagen, CA) master mix, 6 µl of water, 1 µl of each 10 µM primer and 2 µl of eluted DNA. Thermocycler conditions were as follows: 95°C for 15 min; 50 cycles of 95°C for 30 s; 52°C for 30 s; 72°C for 30 s, and a final extension of 72°C for 10 min. We visualized amplicons using 2% agarose 96‐well precast E‐gel (Invitrogen, Life Technologies). We used the PCRClean DX kit (Aline Biosciences) for size selection. We eluted the product in water, and measured the concentration using a Qubit 2.0 spectrophotometer and the Qubit dsDNA HS Assay Kit (Invitrogen, Life Technologies). We normalized the products to 1 ng/µl prior to final library dilution. For sequencing, we used an Ion Torrent platform (Life Technologies) as per Clare et al. (2014) with 192 samples (2 × 96 well plates) in a run using a 316 chip and followed the manufacturers’ guidelines but with a 2× dilution.

The sequences were processed using established methods (Salinas‐Ramos, Herrera Montalvo, León‐Regagnon, Arrizabalaga‐Escudero, & Clare, 2015) which involved demultiplexing reads (allowing two indels and two mismatches in a 10 bp MID), primer adaptor and MID removal, length filtration (157 bp ± 10 bp) and collapsing to unique haplotypes using the Galaxy platform (http//main.g2.bx.psu.edu/root, Afgan et al., 2016. We filtered out singletons and then clustered the remaining haplotypes into molecular operational taxonomic units (MOTUs) at 92%, 94%, and 96% similarity in QIIME using the pick_otu and uclust methods (http://qiime.sourceforge.net/, Caporaso et al., 2010). See a discussion of MOTU thresholds (Clare, Chain, Littlefair, & Cristescu, 2016; Hemprich‐Bennett, Oliveira, Le Comber, Rossiter, & Clare, 2018). We used a BLAST analysis interpreted in MEGAN (Huson, Mitra, Ruscheweyh, Weber, & Schuster, 2011) to filter out MOTUs that could not be reliably classified to Order (those returning no result or unassigned classification or those only classified at higher taxonomic levels). The reference database was based on >600,000 COI sequences extracted from Genbank and spanning all known life (primarily arthropods but also including bacteria, fungi, rotifers etc. for exclusion purposes). We then filtered out sequences thought to be chimeras using UCHIME as implemented in MOTHUR (Schloss et al., 2009). We generated an interaction matrix for each bat and its prey where each cell value represents an observed interaction between a pair (bat and MOTU) where one interaction represents the DNA of a MOTU in the feces of an individual bat (coded 0 and 1). We then calculated the interaction frequency as the number of MOTU in each order found in the fecal samples of an individual bat and then summed for the bat species.

### 2.3 Statistical analyses and dietary composition

We classified all 17 bat species according to their foraging behavior as described by Ratcliffe et al. (2006). Briefly, we used morphological and behavioral data (Norberg & Fenton, 1988; Norberg & Rayner, 1987; Wilson & Reeder, 2005, all available Mammalian Species Accounts) to assign each bat species to one of four foraging behavior categories. Category 1 (ground gleaning bats) consists of ground gleaning predatory bat species as defined by Norberg and Fenton (1988). These bats tend to take large surface‐bound prey, using prey‐generated sounds for the detection and localization of prey. Wing morphology corroborates these observations (Norberg & Rayner, 1987). Category 2 (behaviorally flexible bats) comprises species reported to both glean and hawk prey categorized as gleaning and hovering species or slow hawking species (Norberg & Rayner, 1987). Category 3 (clutter‐tolerant aerial hawking bats) comprises species reported to aerially hawk prey categorized as slow hawking species (Norberg & Rayner, 1987). These species have not, to our knowledge, been reported to glean. Category 4 (open space aerial hawking bats) comprises species reported only to aerially hawk prey in open spaces categorized as fast hawking species by (Norberg & Rayner, 1987).

All analyses were performed for the 92% MOTU dataset, and a subset was repeated using the 94% and 96% MOTU datasets. All statistical analyses and visualizations were performed using R (“R Development Core Team: R: A language and environment for statistical computing,” 2008). We calculated niche overlap between bat species pairs using Pianka's measure of niche overlap following Razgour et al. (2011) for the 92% overlap only. We took the bats’ reported peak echolocation frequency from the literature (Table 1).

For all three MOTU analyses, we measured prey richness and extrapolated dietary richness using Shannon and Simpson indices in the VEGAN package (Oksanen et al., 2017). We compared these Shannon and Simpson estimates of dietary diversity with peak echolocation frequency using a Pearson's correlation coefficient.

For all three MOTU analyses, we performed an ANOSIM on a Bray–Curtis matrix to investigate whether diet differed between behavioral categories. A SIMPER test was then performed to determine which orders contributed to that difference. We visualized the contribution of each order to the observed diet using behavioral category as a predictor and a non‐metric multidimensional scaling (NMDS) ordination. We produced an ordination plot to visualize the result of this scaling in two dimensions. We excluded a single *Myotis ciliolabrum* individual that consumed only a single dietary item and thus caused difficulty in visualization. This single exclusion did not alter the NMDS results.

## 3 RESULTS

### 3.1 Sequencing success, recovery of MOTUs, and dietary richness

**Figure 3:**
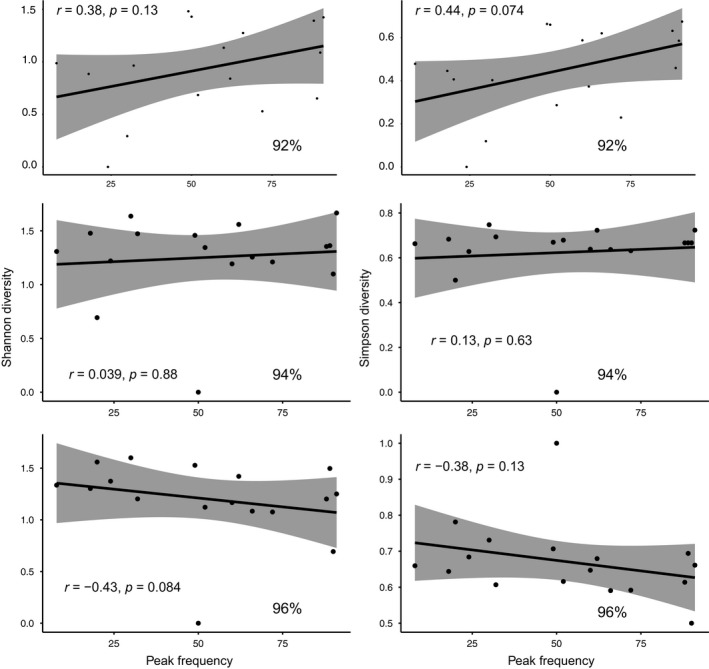
The relationship between echolocation peak frequency and dietary diversity measured using the Shannon and Simpson measurements is not statistically significant for bats in Big Bend National Park Texas. Analyses are performed at 3 MOTU thresholds (92%, 94% and 96%) without any significant effect of MOTU on outcome.

To measure dietary niche breadth among species, we successfully sequenced DNA from 269 samples of the original 309 bats. From an original ≈6.2 million reads, ≈1.3 million were retained post filtering which represent 388,101 haplotypes and 97,896 after singletons were removed. After sequence processing, the 92% MOTU dataset generated a total of 467 MOTUs which were assigned by BLAST to one of 16 arthropod orders (MOTU removed = 1 Chiroptera, 2 parasites, 328 assigned to a higher taxonomic levels, 71 unassigned, 33 no significant BLAST hit, 4 suspected Chimeras). Diptera and Lepidoptera were by far the most diverse prey orders represented by 75% of all MOTUs and both were consumed by all but one of our 17 study species. *Eumops perotis *was the only species that ate Odonata, the least diverse group of insects in the diets of the bats we studied. Chordeumatida, Oribatida, Psocoptera, and Trombidiformes were all found in feces from just one individual per species (8 of 17 species), if present at all. *Myotis thysanodes* (*N* = 32) consumed the highest diversity of prey with MOTU identified to fourteen different insect orders (Table 2). In contrast MOTUs identified in the diet of *Myotis californicus* (*N* = 13) represented only 5 orders. Interestingly, recovery of new taxa was not related to sample size. In *Antrozous pallidus* (*N* = 26), we detected only seven orders all of which were remarkably low in MOTU count (Table 1) while *Eptesicus fuscus* (*N* = 3) consumed ten different insect orders. Observed MOTU richness was not significantly correlated with peak echolocation frequency (Figure 3). As with a 92% MOTU cutoff, observed MOTU richness at 94% and 96% was not significantly correlated with peak echolocation frequency using the Shannon and Simpson diversity estimate. The data were not normally distributed, and thus, the test was performed using a Spearman Rank correlation (Figure 3).

### 3.2 Niche overlap among species

To assess the degree of overlap between species, we performed 66 pairwise comparisons of Pianka's measure of niche overlap using the 92% MOTU dataset, 35 were found to be above 0.6 which indicates that overlap in resource use is high compared to similar systems (Pianka, 1974). Indeed, all 66 values were >0.5 suggesting considerable shared use of resources within the community. The lowest level of overlap was between *Eumops perotis* and *Corynorhinus townsendii* (*O_jk_* = 0.5188) while the highest level of overlap was between *Myotis ciliolabrum* and *Nyctinomops femorosaccus* (*O_jk_* = 0.8309).

### 3.3 Behavior, echolocation and diet

**Figure 4:**
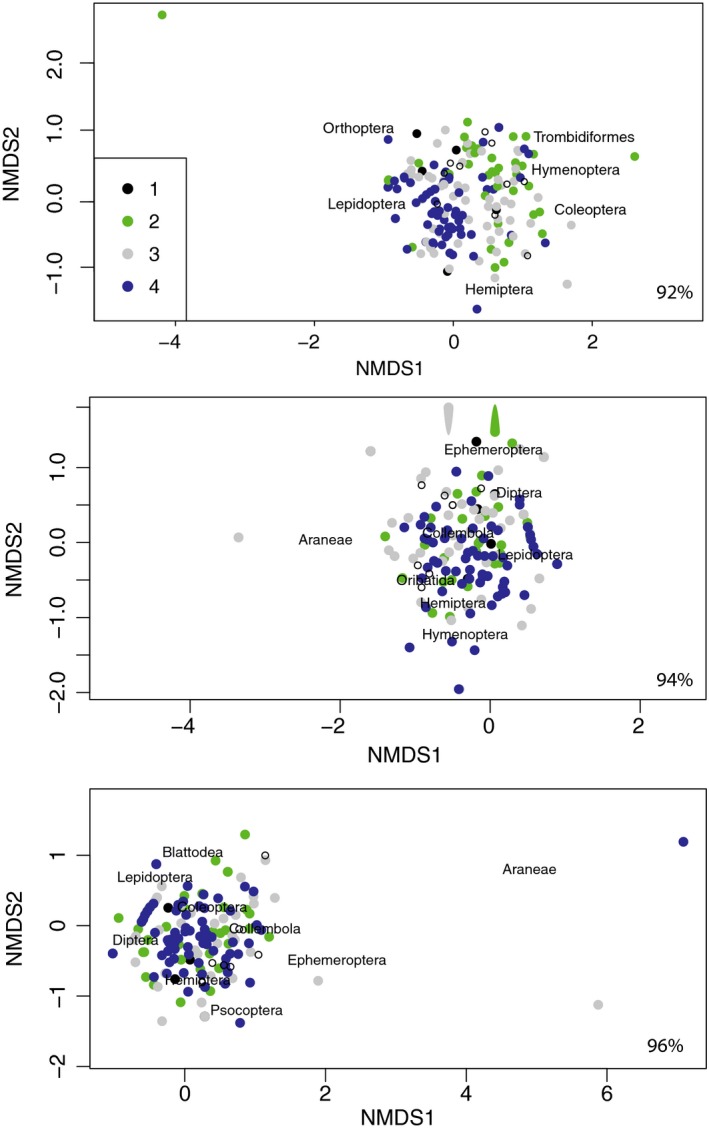
Non‐metric multidimensional scaling (NMDS) of foraging data for bats in Big Bend National Park Texas based on behavioral categories described by Ratcliffe et al. (2006): (1) gleaning bats, (2) behaviorally flexible bats (gleaning and aerial hawking, (3) clutter‐tolerant aerial hawking bats, and (4) open space aerial hawking bats. White dots with black circles indicate prey types with the most common labeled. A SIMPER analysis suggests that the largest difference in prey usage is found between group 2 and 3 bats. Similar outcomes are seen at 3 MOTU thresholds (92%, 94% and 96%)

In an assessment of the relationship between niche, behavior, and echolocation using the 92% MOTU dataset, we found behavioral category was a small but significant predictor of diet (ANOSIM on Bray–Curtis similarity matrix, *R* = 0.1401, *p* = 0.001). A similarity percentage‐species contributions test (SIMPER) indicates that behavioral groups 2 and 3 differed the most in diet, with significant differences in their use of Diptera, Coleoptera, Ephemeroptera, Araneae, Hymenoptera, Trichoptera, Collembola, and Chordeumatida. An NMDS ordination plot showed segregation between behavioral groups (Figure 4). As with the 92% MOTU cutoff, using 94% and 96% cutoffs we found that behavioral category was a small but significant predictor of diet (ANOSIM on Bray–Curtis similarity matrix, 94%, *R* = 0.04465, *p* = 0.01, 96%, *R* = 0.05417, *p* = 0.002). A similarity percentage‐species contributions test (SIMPER) at 94% cutoff indicates that behavioral groups 1 and 3 and 2 and 3 differed the most in diet. A similarity percentage‐species contributions test (SIMPER) at 96% cutoff indicates that behavioral groups 4 and 3, 2 and 3 and 1 and 4 differed the most in diet and an NMDS ordination plot showed minimal segregation between behavioral groups (see Figure 4).

## 4 DISCUSSION

We observed moderate to high niche overlap between pairs of species. Overall, dietary diversity was not related to echolocation call peak frequency but our data suggest that major behavioral categories do predict prey resource use, although the magnitude of the effect is not large. The biggest effect was between those bats that are clutter tolerant but not known to glean, and those that are behaviorally very flexible. Our data support the view that the community is dominated by a pattern of resource sharing and dietary overlap, but that patterns of resource use may be more apparent when measured at the community level and related to broad behavioral strategies, rather than at the species level or by specific hunting or species‐specific characteristics.

### 4.1 Behavior and echolocation strategies of bats in Big Bend National Park

The 17 species of bats included in our statistical analysis can be categorized based on their hunting behavior and echolocation call peak frequency. There is a well‐established relationship between echolocation frequency and object/target resolution (Jones & Teeling, 2006). Among most vertebrates, larger body sizes are associated with the detection, capture, and consumption of both large and small prey, though it has been suggested that this does not apply to bats where perceptual ability is the limiting factor (Barclay & Brigham, 1991). We are not able to measure prey size in our data; thus, we cannot directly test this theory. Our data suggest a trend toward a higher diversity in bats with higher peak frequency, as might be expected if they gain a perceptual advantage to find and track smaller prey, but the relationship is not significant. There are a number of other factors which may be involved in this relationship. Maximum prey size captured may be a function of the bats’ body size and prey handling times (Jakobsen, Brinkløv, & Surlykke, 2013; Jakobsen, Ratcliffe, & Surlykke, 2013). Smaller bats using higher or more broadband frequencies may perceive a larger resource of prey but not be able to handle bigger, harder prey. As such their fundamental niche may be large but their realized niche more limited. For example, we could hypothesize then that *Eumops* may not be able to perceive small prey, while *Myotis* may be able to perceive large and small prey but not handle the large prey. A third dynamic may be flight performance which is difficult to separate from echolocation call structure but may be very closely associated to prey capture success (Norberg & Rayner, 1987) and thus individual niches. Carefully constructed experimental designs would be required to tease apart these factors.

We observed that hunting behavior is a significant predictor of diet, although the variation explained is small. In particular, species that are behaviorally flexible (e.g., *Corynorhinus townsendii, *which gleans but can also hawk prey) differed from clutter hawking species (e.g., most *Myotis *spp.). In our analysis, only *C. townsendii* and *Myotis thysanodes* fell into the behaviorally flexible guild but both had very high taxonomic richness of their prey (ranking 4th and 1st respectively) and, while the SIMPER analysis suggests they differ most in their use of orders from the diet of clutter‐tolerant species, the NMDS suggests that the other guilds are characterized as consuming a subset of the prey of these two species. This suggests that the behaviorally flexible guild consumes the most diverse diet which has substantial bearing on their resilience to unstable environments (Clare et al., 2018). One limitation to our collection is the longitudinal nature of the samples. Many of these bat species are hard to catch and thus our samples span a number of field seasons and years. This introduces a temporal variable to the data which we cannot easily test for but should be kept in mind when interpreting our data. Ideally, all samples would be collected in one concerted effort, but this is impractical in this location (for example only three *Euderma* have ever been caught in 22 years of surveys (Ammerman, personal observation)).

The ability to glean prey from terrestrial surfaces (e.g., vegetation) or not (as in most *Myotis* and all molossids) even when hunting in similarly cluttered habitat, may explain differences in prey. We did not detect any significant segregation of bats using the gleaning niche; however, recent studies have shown a high degree of variation in the diet of bats using this approach, in addition to their acute hearing, these species almost certainly take flying insects as well (Hackett, Korine, & Holderied, 2014; Roswag, Becker, & Encarnac, 2018). This indicates that the gleaning approach does not lead to a novel niche but simply a broader niche. Indeed this approach may be a significant contributor to prey captures and avoiding prey defences (Clare & Holderied, 2015). In this case, the gleaning niche may be one of spatial variation rather than taxonomic.

However, this interpretation should be treated with caution. The primers used to amplify DNA are known to be very broad spectrum but do show taxonomic preferences (Alberdi, Aizpurua, Gilbert, & Bohmann, 2018) and spiders and ants are notoriously difficult to amplify in some mixed DNA templates even when using primers which target them specifically. In our analysis, we did not detect large amounts of Araneae across any species and did not detect scorpions in the diet of *Antrozous*. Despite the fact that recent work has shown that *Antrozous* is resistant to the venom of scorpions (Hopp, Arvidson, Adams, & Razak, 2017) it appears to be a minor component of their diet (Bell, 1982; Johnston & Fenton, 2001; O'Shea & Vaughan, 1977). Obligate gleaning is a relatively rare approach to hunting and only one species in this analysis (*Antrozous pallidus*) could possibly be categorized this way. Our failure to detect a unique gleaning niche may reflect the low incidence of species in this category and thus a dataset leaving us with little power to detect the niche. In any case, our result for *Antrozous* should be treated cautiously but with interest.

In this analysis, we employed a MOTU cutoff of 92% and repeated some key analyses at 94% and 96%. This is consistent with many previously published studies which have used a wide variety of thresholds (Alberdi et al., 2018; Pearson et al., 2018; Salinas‐Ramos et al., 2015) but is lower than standard barcode divergences reported for arthropods (e.g., Hebert, Cywinska, Ball, & DeWaard, 2003). One common problem in metabarcoding data is MOTU inflation driven by sequencing error (Flynn, Brown, Chain, MacIsaac, & Cristescu, 2015). While MOTU were never meant to represent species and in the original description (Floyd, Abebe, Papert, & Blaxter, 2002) it was argued that they “need not correspond to identity of operational taxonomic units (OTU) as measured by other models (biological or morphological)” the correspondence of specific cutoffs to taxa and their relevance to taxonomic levels are continually debated (e.g., Zeale et al., 2011). Because of the variability in sequence divergence between species across taxa, highly diverse taxonomic assemblages (e.g., arthropods) cannot be characterized by a single sequence threshold. As such MOTU are best thought of as pools of equal genetic diversity rather than any specific taxonomic rank.

A reasonable question, then, is what impact do specific thresholds have on ecological models? An extensive test of the impact of the clustering level on Pianka's niche test has been conducted on two separate occasions. Clare et al. (2016) tested 176 different combinations of bioinformatics clustering, filtering, and demultiplexing parameters in an almost identically produced dataset and assessed the impact on Pianka's ecological niche model. This is the most extensive such test of ecological impact of bioinformatics steps we know of and the analysis showed the conclusions were robust. Given the outcome, and the risk of MOTU inflation, conservative clustering was recommended (Clare et al., 2016). Similarly Salinas‐Ramos et al. (2015) performed similar ecological analyses of bat diet data on datasets clustered at 92%, 94%, and 96% cutoffs and similarly demonstrate no difference in the conclusions. To this, we have added an analysis of our correlation and NMDS at 94% and 96% (Figures 3 and 4). We find minimal impact in all measures. Our use of 92% is not arbitrary but was empirically established (Salinas‐Ramos et al., 2015) to minimize the obvious cases of MOTU over‐inflation when using these primers and this sequencing platform.

Other cutoffs have been used (e.g., 89% Pearson et al., 2018, 94%, Clare et al., 2014) in similar analyses; thus, there are a variety of used parameters but all evidence suggests the effect is minimal and, in general, conservative values are preferred. While we only provide identifications to Order as would be expected from morphological analysis, molecular approaches are known to identify small soft‐bodied prey with increased accuracy (Clare, Fraser, Braid, Fenton, & Hebert, 2009) and the MOTU approach provides a quantification of the diversity of the orders in this analysis. It would be possible to identify some of the raw sequences to species using a comparison to a reference database though this introduces two biases, one in favor of species whose DNA is less likely to degrade during digestion and one in favor of species which are more likely to be found in reference collections (e.g., larger more charismatic or economically important species). This can provide names for interest but it is not advisable to analyze those data (e.g., Littlefair, Zander, de Sena Costa, & Clare, 2018). For an extensive discussion of the use of names versus MOTU and introduced biases see Clare et al. (2018). Another factor to consider is that over the course of our study two preservation methods were used. Most samples were desiccated and frozen however some were stored only in ethanol. The main reason for using ethanol is to prevent fungal and bacterial growth that can overwhelm samples that have not been frozen. That said, we have previously used both preservation methods and observed no difference in success rates for the analysis of insect DNA (Clare personal observation) thus it is unlikely to be a factor in this analysis.

### 4.2 Theories of niche use

Niche theory predicts that rare or unpredictable resources increase competition (Hardin, 1960). Pianka found desert systems produce niche segregation (Pianka, 1974) and this has been attributed to reduced or unpredictable resources. In assessments of resource use, diet is frequently a key indicator for competitive relationships among consumers which may drive niche segregation; however, our data suggest considerable niche overlap. Tebbich, Taborsky, Fessl, Dvorak, and Winkler (2004) reported an increase in diet flexibility of finches when resources were low in abundance. Similar findings in other bat species have suggested niches may broaden when resources are reduced (e.g., Clare et al., 2014; Razgour et al., 2011; Salinas‐Ramos et al., 2015). In contrast to niche theory, optimal foraging theory predicts that when food resources are limited, dietary breadth will increase due to foraging and selectivity becoming more costly and that this may generate more subtle patterns of resource use as we observe here (Vesterinen et al., 2016).

While we looked at categories of behavior as discrete, it is important to note that many insectivorous bats are known to be behaviorally flexible (e.g., Ratcliffe & Dawson, 2003), particularly when resources are limited (Clare et al., 2014; Razgour et al., 2011) as could be expected in a desert environment (Pianka, 1973). Low frequency echolocators are better suited to open spaces with low clutter, while high frequency broadband echolocators are more likely to hunt in edge habitats where the calls do not need to travel as far and resolution is important (Fenton, 1990; Neuweiler, 1984, 1989; Schnitzler & Kalko, 2001) but when an environment is not well described by distinct and discrete zones this may become a somewhat artificial division.

In our analysis, we found evidence that, at the community level, foraging behavior is associated with resources use in co‐occurring species but that it is subtle. Thus, we suggest that principles of both niche theory and optimal foraging may help us to understand to the structure of this community. Previous work has found only minimal evidence of niche partitioning in bats (Arrizabalaga‐Escudero et al., 2018; Matthews, Neiswenter, & Ammerman, 2010; Razgour et al., 2011; Salinas‐Ramos et al., 2015); however, almost all previous work has been limited to a small subset of the community (though see for example Emrich et al., 2014; Galan et al., 2018). Here, we have taken a novel approach of considering partitioning at the level of the community and as such we can analyze categories of behavior rather than species in isolation and we observe that partitioning is more apparent or more detectable at this level. This suggests that some ecological structure is found at the level of the community that is not at the level of individual species (Schnitzler & Kalko, 2001).

A number of additional mechanisms of resource partitioning may not be easily detected here. Sympatric bats in Jamaica may avoid direct inter and intraspecific competition by segregating the insect resource in time (Emrich et al., 2014). Radio tracking of European bats has shown that hunting times differ between cryptic bat species (Nicholls & Racey, 2006). This tactic allows even morphologically distinct species to consume the same prey, and therefore have almost identical dietary richness and very high dietary overlap while still partitioning the resource. In our analysis of niche overlap, *Myotis ciliolabrum* and *Nyctinomops femorosaccus* showed high dietary overlap while *Eumops perotis* and *Corynorhinus townsendii* are highlighted as having the least overlap in diet. Both *M. ciliolabrum* and *N. femorosaccus* are smaller bats that likely take flying prey in fairly open habitat and thus a strong dietary overlap in two distantly related bats is not surprising. In the second case, *E. perotis* is large and flies fast in open space and is very unlikely to glean, while *C. townsendii* both gleans and hawks and can do so in small spaces. Thus, they may overlap little in spatial use and foraging behavior and our dietary analysis reflects this. Fine grained spatial segregation of foraging is also a recognized mechanism among rhinolophid bats in southwest Iberia (Spain) where sympatric sibling species, partitioned resources in space, rather than in time (Arrizabalaga‐Escudero et al., 2018; Salsamendi, Garin, Arostegui, Goiti, & Aihartza, 2012) suggesting that strong competitive pressure has led to spatiotemporal partitioning but little difference in actual dietary makeup. Such fine grained segregation has also been observed among bats in Madagascar (Dammhahn & Goodman, 2014) and a similar effect may be at play in this Texas bat community. Our focus here is on diet, but alternative mechanisms of co‐existence and explanations for species‐rich communities are key in these communities. In particular, the availability of roosting sites is often a strong predictor of species richness (Kunz & Fenton., 2005; Willis et al., 2006) and deserts may be uniquely rich in crevices and caves and thus support unexpected species richness.

We analyzed a single dropping from *Euderma maculatum* that contained only Lepidoptera (Table 2). *E. maculatum* is a known moth specialist (Ammerman et al., 2012) which is confirmed here. One particularly interesting observation was the presence of Odonata exclusively in the diet of *Eumops*. Because of their long narrow wing shape, *Eumops* cannot take off from a surface and thus cannot glean (Ammerman et al., 2012). In order to gain lift, *Eumops* roost in rock crevices above an unobstructed vertical drop (Ammerman et al., 2012). Although they emerge late in the evening, crickets and dragonflies have been reported as prey before and some have suggested they take these prey from the rock walls near their roosts (Ammerman et al., 2012). Additionally, *Eumops* large body size, fast flight and low peak frequency may allow for the long‐range, fast‐paced tracking of large insects, like dragonflies, if this bat's foraging activity ever overlaps with that of these insects.

## 5 CONCLUSIONS

Here, we provide one of the first analyses of a community of insectivorous bats using metabarcoding that includes nearly all species and not just a small subset. We detect structure in the use of resources at the level of the community that are not easily detected at the level of individual bat species. Our analysis suggests that hunting behavior is associated with resource divisions in this community and that echolocation peak frequency may be involved but the effect is not strong. These data suggest that patterns of resource use may not be obvious at the level of individual species of bat but that at the community level patterns are more distinct.

## CONFLICT OF INTEREST

None declared.

## AUTHOR CONTRIBUTIONS

ELC, LA, JMR, and MBF performed fieldwork. RG, SI, and JE performed laboratory work. RG, SI, JE, ELC, and JMR performed analyses. All authors contributed to the writing of the manuscript.

## Data Availability

Sequence read files can be found in the Dryad database (https://doi.org/10.5061/dryad.7j0c8dm).
